# Gene Expression Analysis Provides Insights Into the Functional and Developmental Differentiations of Pleopodal Lungs in a Terrestrial Isopod Crustacean, *Porcellio scaber*

**DOI:** 10.1111/ede.70026

**Published:** 2025-11-29

**Authors:** Naoto Inui, Akifumi Yao, Sumio Udagawa, Kohei Oguchi, Yoshinobu Hayashi, Toru Miura

**Affiliations:** ^1^ Misaki Marine Biological Station, School of Science The University of Tokyo Miura Kanagawa Japan; ^2^ National Institute of Genetics Shizuoka Japan; ^3^ Tateyama Marine Laboratory, Marine and Coastal Research Center Ochanomizu University Tateyama Chiba Japan; ^4^ Department of Biology Keio University Yokohama Japan

**Keywords:** appendage, gill, morphogenesis, respiratory organ, RNA‐seq, terrestrialization

## Abstract

Acquisition of air‐breathing organs was one of the key events in the diversification of arthropods. Among terrestrial arthropods, isopod crustaceans have evolved a unique air‐breathing structure called the pleopodal lung, which is located in their abdominal appendages (pleopods), while retaining pleopodal gills. These lungs offer an intriguing model for studying the evolution of respiratory organs during arthropod terrestrialization. However, the molecular mechanisms underlying lung function or development in isopods remain poorly understood. In this study, we conducted comparative transcriptomic analyzes using the common rough woodlouse, *Porcellio scaber*, in which pleopods with and without lungs are adjacent to each other. The results revealed distinct gene expression profiles linked to the structure and function of pleopods, including genes involved in morphogenesis. In particular, candidate lung development regulatory genes that were expressed specifically in the exopods of the second pleopods during the manca 1 stage were identified. Transcriptome analysis and immunohistochemistry suggested that the Hox gene *abdominal‐A* is involved in lung formation. However, the two genes previously implicated in respiratory organ formation in pancrustaceans, *trachealess* and *ventral veins lacking*, did not show lung‐related expression. Our comparison of gene expression patterns between exopods with and without lungs suggest that the function of gas exchange in the pleopodal lungs may be influenced by structural differences resulting from changes in developmental processes. Overall, this study provides essential insights into the molecular mechanisms underlying pleopodal lung development and sets the foundation for future evolutionary research.

## Summary

Pleopodal lungs are novel air‐breathing organs acquired in terrestrial isopod lineages. In this study, transcriptomic analyzes were performed on different types of pleopods, namely lung‐bearing exopods, normal exopods and endopods in a terrestrial isopod. Possible candidate genes involved in pleopodal lung development were identified.

## Graphical abstract

Transcriptomic analysis of pleopods in *Porcellio scaber* reveals gene expression linked to pleopodal lung development, identifying candidate regulatory genes and highlighting developmental changes as drivers of lung development in terrestrial isopod crustaceans.

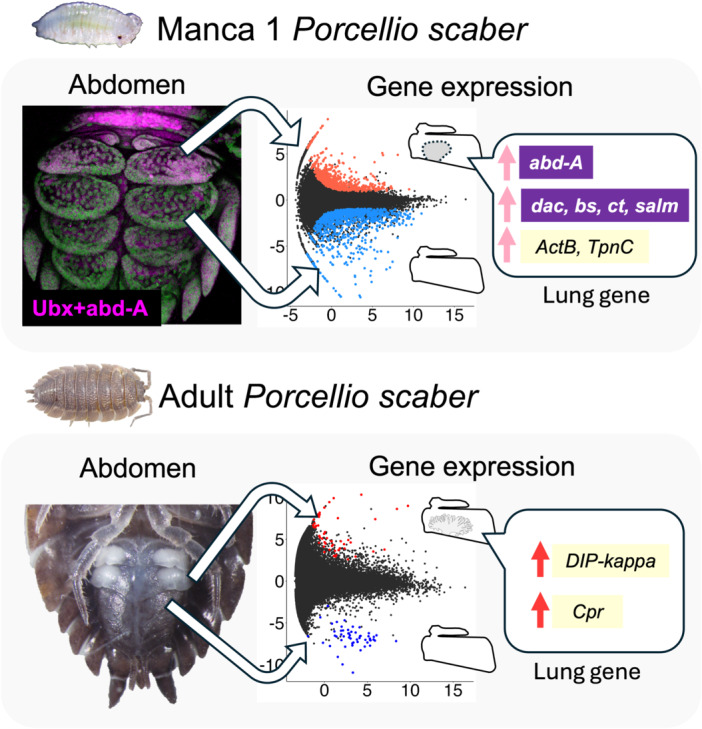

## 1 Introduction

The transition from aquatic to terrestrial life is a pivotal event in animal evolutionary history. Among various animal groups, arthropods have achieved remarkable success on land, with the highest number of terrestrial species. This success is partly attributed to the evolution of specialized respiratory structures that enable air breathing (Hsia et al. 2013; Sharma 2017). Different arthropod lineages have developed distinct respiratory adaptations: insects, myriapods, and some terrestrial chelicerates possess a tracheal system, while some terrestrial chelicerates and terrestrial crustaceans have evolved lung‐like organs (Hsia et al. 2013; Sharma 2017; Tan and Monteiro 2025).

The acquisition of air‐breathing organs in terrestrial arthropods is thought to have occurred independently in different lineages (Hsia et al. 2013; Sharma 2017). However, key transcription factors involved in tracheal development in insects, namely *trachealess* (*trh*) and *ventral veins lacking* (*vvl*), are also expressed in the gills of crustaceans and the respiratory organs of arachnids (Franch‐Marro et al. 2006; Medina‐Jiménez et al. 2024; Sharma 2017). These findings suggest that *trh* and *vvl* may play a broader role in the development of respiratory structures in arthropods or pancrustaceans (Bruce 2021; Sharma 2017). Furthermore, it has been proposed that the origins of respiratory organs may trace back to a common ancestor (Franch‐Marro et al. 2006). Given this background, arthropod respiratory structures are good models for obtaining insights into the convergence and divergence of adaptive traits.

Among terrestrial arthropods, isopod crustaceans represent a unique lineage due to the diversity of their respiratory structures (Boxshall and Jaume 2009; Hornung 2011; Tan and Monteiro 2025). Their abdominal appendages, known as pleopods, function as respiratory organs (Boxshall and Jaume 2009). In terrestrial isopods, specialized air‐breathing structures develop in the pleopods through epithelial morphogenesis during postembryonic stages (Hornung 2011; Inui et al. 2022). However, the molecular mechanisms underlying the formation of these structures remain largely unknown. Notably, previous studies showed that *vvl* is expressed in the posterior pleopods of terrestrial isopod embryos, but not specifically in air‐breathing structures (Abzhanov and Kaufman 2000b). In addition, it has been suggested that Hox genes and some transcription factors responsible for appendage patterning could be involved in the morphogenesis of isopod pleopods and air‐breathing structures (Abzhanov and Kaufman 2000b; Inui and Miura 2023; Tan and Monteiro 2025). Investigating the molecular basis of these isopod respiratory adaptations offers valuable insights into the evolution of arthropod respiratory organs (Tan and Monteiro 2025).

**Figure 1:**
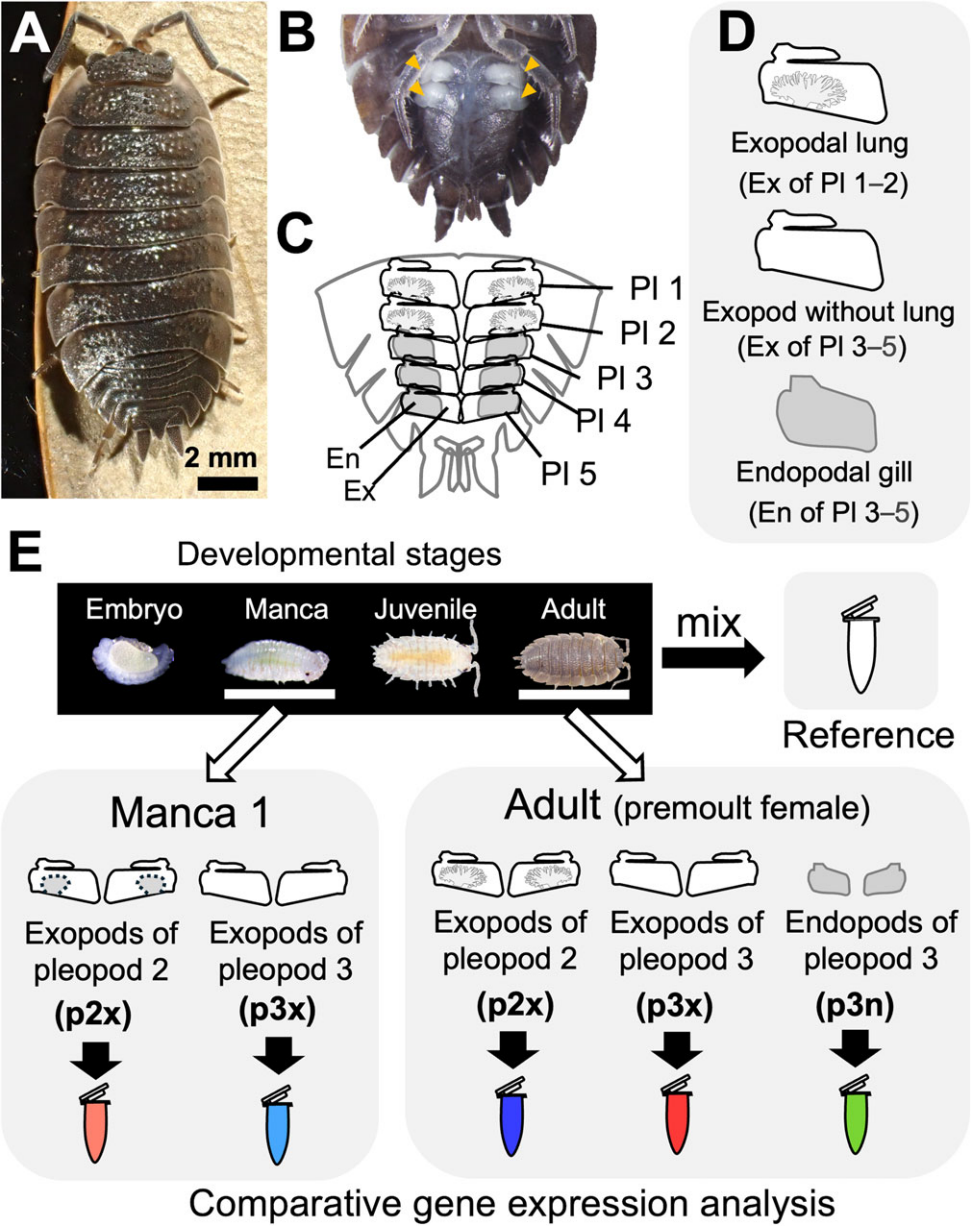
Experimental design for transcriptomic analysis. (A) A photograph of the study species, isopod *Porcellio scaber*. (B) Ventral view of the abdomen (adult female). The seventh pereopods were removed. Yellow arrowheads indicate pleopodal lungs. (C and D) Schematic diagram of isopod abdomen (adult female) and five pairs of pleopods. Pleopods bifurcated into exopods and endopods. Exopods of the first two pleopods develop lung structures. In males, endopods of the anterior pleopods function as copulatory organs, but females lack endopods in anterior two pleopods. Endopods of the posterior pleopods function as gills. (E) RNA samples for sequencing. An RNA sample extracted from whole body of various developmental stages was sequenced for the reference transcriptome. Two kinds of pleopods at a manca stage and three kinds of pleopods at the adult stage were examined for gene expression analysis. Samples for comparative analysis included three biological replicates (Supporting Information Table S1). En, endopod; Ex, exopod; Pl, pleopod; p2x, the exopods of the second pleopods; p3n, the endopods of the third pleopods; p3x, the exopods of the third pleopods. [Color figure can be viewed at wileyonlinelibrary.com]

To investigate the mechanisms underlying air‐breathing structure development in isopods, a comprehensive gene expression analysis could be an effective initial approach. Therefore, here we performed transcriptomic analyzes to examine gene expression patterns associated with air‐breathing structure development in the terrestrial isopod *Porcellio scaber*. In this species, only the anterior two pairs of pleopods develop air‐breathing structures (pleopodal lungs), while the posterior pleopods retain their ancestral forms: the cover‐like exopods and endopodal gills (Figure 1A–D). Although the endopodal gills of terrestrial isopods may also have gas‐exchange abilities, they have osmoregulatory function in a water chamber covered by exopods, suggesting that they are functionally different from lungs (Tan and Monteiro 2025). We compared gene expression between the exopods of pleopod 2 (lung‐bearing) and pleopod 3 (non‐lung) at two developmental stages: the manca 1 stage (Inui et al. 2022) and adult. Additionally, gene expression patterns in the adult endopods of pleopods were examined for further comparison. The expression patterns of morphogenetic factors involving lung development were investigated and immunohistochemistry was performed for Hox genes. Based on these analyzes, the developmental mechanisms underlying functional diversification in isopod pleopods are discussed.

## 2 Materials and Methods

### 2.1 RNA Extraction

At first, to construct a reference transcriptome assembly of *P. scaber*, total RNA was extracted at various development stages of the focal species, including embryos, mancae, juveniles and adults. *Porcellio scaber* were prepared according to the previous report (Inui et al. 2022). Stagings were defined according to previous studies (Drobne et al. 1998; Inui et al. 2022). RNA extraction was carried out using ISOGEN (Nippon Gene, Tokyo, Japan), and the extracted RNA was treated with DNase I (Thermo Fisher Scientific, MA, USA) and purified using RNA Clean XP beads (Beckman Coulter Genomics, MA, USA). RNAs extracted from the samples were then pooled together (Figure 1E).

Next, to compare the gene expression among different kinds of pleopods containing developing lungs, the following biological groups were selected for comparative transcriptomic analysis: the exopods of the second and third pleopods for manca 1 (Inui et al. 2022), the exopods of the second pleopods in premoult substage 2 female adults, and the exopods and endopods of the third pleopods in premoult substage 2 female adults (Drobne et al. 1998) (Figure 1E). Three biological replicates were prepared for each group.

For manca samples, 90 pairs of pleopods were dissected in RNAlater (Ambion Inc., TX, USA) and pooled for one sample. For adult samples, 4 pairs of pleopods were dissected and pooled for one sample. After dissection, total RNA was extracted using a ReliaPrep RNA Tissue Miniprep Kit (Promega, WI, USA) according to the manufacturer's instructions. The quality and quantity of extracted RNA samples were checked using NanoDrop One^C^ (ThermoFisher Scientific, MA, USA), Qubit RNA HS Assay system (ThermoFisher scientific, MA, USA) and 2100 Bioanalyzer system (Agilent Technologies, CA, USA).

### 2.2 Library Preparation and Sequencing

The pooled RNA sample for the reference transcriptome was firstly sequenced. Library preparation and sequencing were performed by Eurofin Genomics (Tokyo, Japan). A strand‐specific mRNA sequencing library was constructed from the pooled total RNA. Paired‐end sequencing in an Illumina Novaseq 6000 platform (Illumina, CA, USA) was conducted to produce 150‐bp reads from both ends of each DNA fragment.

As the amount of RNA obtained varies depending on the stage (manca: 20–30 ng/sample; adult: 250–500 ng/sample), RNA sequencing for comparative expression analysis was performed using different methods between the adult and manca samples.

For manca samples, the total RNA samples were sent to the biotechnology company Genome‐Lead Inc. (Kagawa, Japan) for processing. The libraries were prepared by using an MGIEasy RNA Directional Library Prep Set (MGI Tech, Guangdong, China) with total RNA enrichment. DNA nanoballs were prepared from the libraries using a DNBSEQ‐T7RS High‐Throughput Sequencing Set (MGI Tech). DNA nanoball libraries were sequenced on a DNBSEQ‐G400RS sequencer (MGI Tech) with the 2 × 150‐bp paired‐end sequencing protocol.

For adult samples, the cDNA libraries were prepared by the author using the NEBNext Directional Ultra II RNA Library Prep Kit (New England BioLabs Ltd., MA, USA) with messenger RNA (mRNA) enrichment (NEBNext Poly(A) mRNA Magnetic Isolation Module). Then the libraries were sent to the biotechnology company Azenta Life Sciences Japan Inc. (Tokyo, Japan) for sequencing. DNA libraries were sequenced on the Illumina NovaSeq X Plus sequencer system (Illumina, CA, USA) with the 2 × 150‐bp paired‐end sequencing protocol.

All reads were deposited in the DDBJ Sequence Read Archive (DRA) database under accession numbers DRR640357–DRR640372 (BioProject accession number: PRJDB20244).

### 2.3 Constructing Transcriptome Assembly

Before assembling the sequencing reads, sequences of the adapters used for Illumina sequencing and low‐quality bases were trimmed from the sequencing reads using Trimmomatic version 0.36 (Bolger et al. 2014). The adapter‐trimmed and quality‐filtered paired reads were then subjected to de novo transcriptome assembly using Trinity version 2.6.6 (Grabherr et al. 2011) with default options. Completeness of assembled sequences was assessed using BUSCO version 5.8.1 (Manni et al. 2021) against the single copy ortholog set of arthropoda_odb10. The transcriptome assembly sequence was deposited in the DDBJ Transcriptome Shotgun Assembly (TSA) database under accession number ICXA01000000 (BioProject accession number: PRJDB20244).

### 2.4 Curating Reference Transcriptome of *P. scaber*

To reduce the redundancy of the reference transcriptome of *P. scaber*, superTranscripts were constructed according to previous studies (Davidson et al. 2017; Yao et al. 2024) using gene expression data of the manca sample. For sequencing of manca samples, read quality was checked by FastQC v. 0.11.9

(http://www.bioinformatics.babraham.ac.uk/projects/fastqc/) and adapter sequences were removed by Trimmomatic v.0.39 (Bolger et al. 2014). The trimmed RNA‐seq reads were firstly mapped to the ribosomal RNA (rRNA) sequences of *P. scaber* obtained from the reference transcriptome and NCBI database (Accession number: AJ287062.1, EU914253.1) using BWA‐mem2 (Vasimuddin et al. 2019) to remove rRNA sequences. The unmapped reads were secondly mapped to the reference transcriptome using salmon version 1.9.0 (Patro et al. 2017). Finally, superTranscripts was constructed using Corset version 1.0.9 and Lace version 1.00 (Davidson et al. 2017).

### 2.5 Gene Annotation of Curated Transcriptome

The constructed superTranscripts were functionally annotated according to the previous study (Yao et al. 2024). To detect protein‐coding sequences, open reading frames (ORFs) in superTranscipts were predicted using TransDecoder version 5.5.0

(https://github.com/TransDecoder/TransDecoder) with “single_best_orf” option and minimum protein length of 50. Completeness of optimized sequences was assessed using BUSCO against arthropoda_odb10. Then protein sequences were submitted to eggNOG‐Mapper (Cantalapiedra et al. 2021; Huerta‐Cepas et al. 2018) and Pannzer2 (Törönen and Holm 2022). Furthermore, reciprocal BLAST best‐hit analysis against nonredundant protein sequences of the model fruit fly *Drosophila melanogaster* was conducted using blastp version 2.16.0 (Altschul et al. 1990) with e‐value 1e‐4 as the threshold. To remove the redundant sequences (isoforms), longest isoforms were obtained from the NCBI RefSeq genome of *D. melanogaster* using retrieve_longest_isoforms() function in the R package orthologr version 0.4.0 (Drost et al. 2015). The superTranscripts and annotation files were deposited in the Zenodo database (doi. org/10.5281/zenodo.15011027).

### 2.6 Expression Quantification

The sequence data obtained from adults were also curated using fastQC and Trimmomatic in the same way as for the manca samples.

For all samples obtained from mancae and adults, curated reads were mapped against the superTranscripts using STAR version 2.7.8a (Dobin et al. 2013), and transcript abundances were estimated using featureCounts in subread version 2.0.6 (Liao et al. 2013) with default settings. In addition, the number of transcripts per million (TPM) was calculated using RNAnorm (Zmrzlikar et al. 2023).

### 2.7 Principal Component Analysis

Principal component analyzes (PCA) were performed to visualize relationships among samples. Due to the difference in the sequencing method, the adult and manca samples were analyzed separately. To avoid the influence of low‐expression transcripts on the results, transcripts with a TPM value of at least 1 in each sample and a sum of all TPM values of at least 10 were analyzed. After logarithmic conversion of each TPM value, the top 3,000 transcripts with greatest variance across samples were used for PCA. PCA and plots were made using R version 4.4.2 (R core team 2024) with the geom_mark_ellipse () function in ggforce version 0.4.2 (Pedersen 2022).

### 2.8 Differential Expression Analysis

Differential expression analyzes were performed using a pairwise comparison framework for manca samples and a generalized linear model (GLM) framework for adult samples in edgeR version 4.4.0 (Y. Chen et al. 2025) in R. Counts were normalized using the trimmed mean of M‐values (TMM) method without low‐expression gene filtering. For each comparison, dispersion estimates were obtained using the empirical Bayes method, and differential expression was assessed using exact tests (pairwise comparison) or quasi‐likelihood F‐tests (GLM). For the GLM analysis, a design matrix was constructed to model the three groups in adult samples, allowing estimation of group‐specific coefficients. *p*‐values were adjusted for multiple testing using the Benjamini–Hochberg method to control the false discovery rate (FDR). The threshold of FDR was set to 0.1 to define significant differential expression, and to detect more possible candidate genes related to lung development, no fold change cutoff was applied.

### 2.9 Gene Set Enrichment Analysis

To identify gene‐expression characteristics associated with biological groups, gene set enrichment analyzes were performed using gseGO() function in clusterProfiler version 4.14.3 (Xu et al. 2024) in R. AnnotationForge version 1.48.0 (Carlson and Pagès 2022) was used to create a custom OrgDB background for *P. scaber* based on annotated superTranscripts. Expression levels of transcripts were based on the logFC values from differential gene expression analysis. Significantly enriched GO terms (*p* < 0.05) for “biological processes” were identified based on the Benjamini–Hochberg adjusted *p*‐value. To reduce GO term redundancy, the simplify() function in clusterProfiler was applied to the results.

### 2.10 Data Integration and Visualization

Data integration and visualization were basically performed using tidyverse version 2.0.0 (Wickham et al. 2019) in R. Venn diagrams were drawn using VennDiagram version 1.7.3 (Chen and Boutros 2011). The heatmap of TPM was drawn using pheatmap version 1.0.12 (Kolde 2018).

### 2.11 Immunofluorescence

To visualize the Hox gene expression in developing lung structures, immunofluorescence was performed. After fixation of the manca 1 stage of *P. scaber* with 4% paraformaldehyde (PFA) in 1X phosphate‐buffered saline (PBS) for 1 h, the fixed sample was washed in PBS with 0.3% Triton X‐100 (PBT) for 15 min at least three times and dehydrated with methanol before staining. To improve antibody penetration, the sample was rehydrated in 0.3% Triton‐X in PBS, sonicated briefly, and treated for 24 h at room temperature with a solution composed of 10% vol/vol ethylenediaminetetraacetic acid trisodium salt dihydrate (EDTA‐3Na) in 1X PBS. Then, the sample was blocked with 0.2% vol/vol bovine serum albumin and 2% vol/vol normal goat serum in PBT for 30 min. After that, the sample was incubated with primary antibody FP6.87 (1:100, Developmental Studies Hybridoma Bank), which recognizes Ultrabithorax (Ubx) and Abdominal‐A (Abd‐A) (Kelsh et al. 1994), for 2 days in a refrigerator. After washing with PBT, the sample was blocked in the same way as for the first incubation and incubated with fluorescent‐conjugated secondary antibodies (Alexa Fluor 555 Goat anti‐Mouse IgG, Life Technologies, CA, USA) for 2 days in a refrigerator. After washing with PBT, the sample was stained with 4′, 6‐diamidino‐2‐phenylindole (DAPI) (2 μg/mL; Sigma) to label cell nuclei. Finally, the sample was washed for 15 min in PBT at least three times, mounted on a glass‐bottom dish, and observed using a confocal microscope (FV3000; Olympus, Tokyo, Japan) with laser excitations of 405 nm (DAPI) and 561 nm (FP6.87 with Alexa 555). Images were processed with software FV31S‐DT (Olympus, Tokyo, Japan) and ImageJ (Schneider et al. 2012).

## 3 Results

### 3.1 Sequencing Results and Reference Transcriptome Assembly

Sequencing data were obtained from 1 sample at various developmental stages, 6 samples at manca 1 stage, and 9 samples at premoult adult stage (Figure 1E: Supporting Information Table S1). To detect mRNA expression, manca samples with total RNA library preparation were sequenced more deeply (208,211,871**–**437,917,442 reads) than adult samples (11,166,824**–**19,695,037 reads). After rRNA removal, the number of reads in manca samples decreased (19,326,303**–**43,065,064 reads) (Supporting Information Table S1).

The *de novo* transcriptome assembly process generated 361,183 contigs from the pooled RNA sample (Supporting Information Figure S3: Table S2). 97.9% (duplicate 61.9%) of complete arthropod BUSCOs (Benchmarking Universal Single‐Copy Orthologs) were present in the reference transcriptome of *P. scaber* (Supporting Information Table S2).

For downstream gene expression analysis, a curated assembly (superTranscripts) was constructed based on the reference transcriptome of *P. scaber*. After clustering, 143,469 superTranscripts were obtained with 27,157 protein‐coding sequences, an N50 of 755 bp (Supporting Information S3; Supporting Information Table S2). 90.9% (duplicate 1.2%) of complete arthropod BUSCOs were present in superTranscripts. The results showed that 10,910 (40.2%) and 7775 sequences (28.6%) of protein coding sequences were annotated by eggNOG‐mapper and panzzer2, respectively. In addition, reciprocal BLAST best‐hit analysis identified 5980 sequences (22.0%). In total, 12,482 sequences (46.0%) were successfully annotated (Supporting Information S3).

### 3.2 Gene Expression Patterns Revealed by PCA

**Figure 2:**
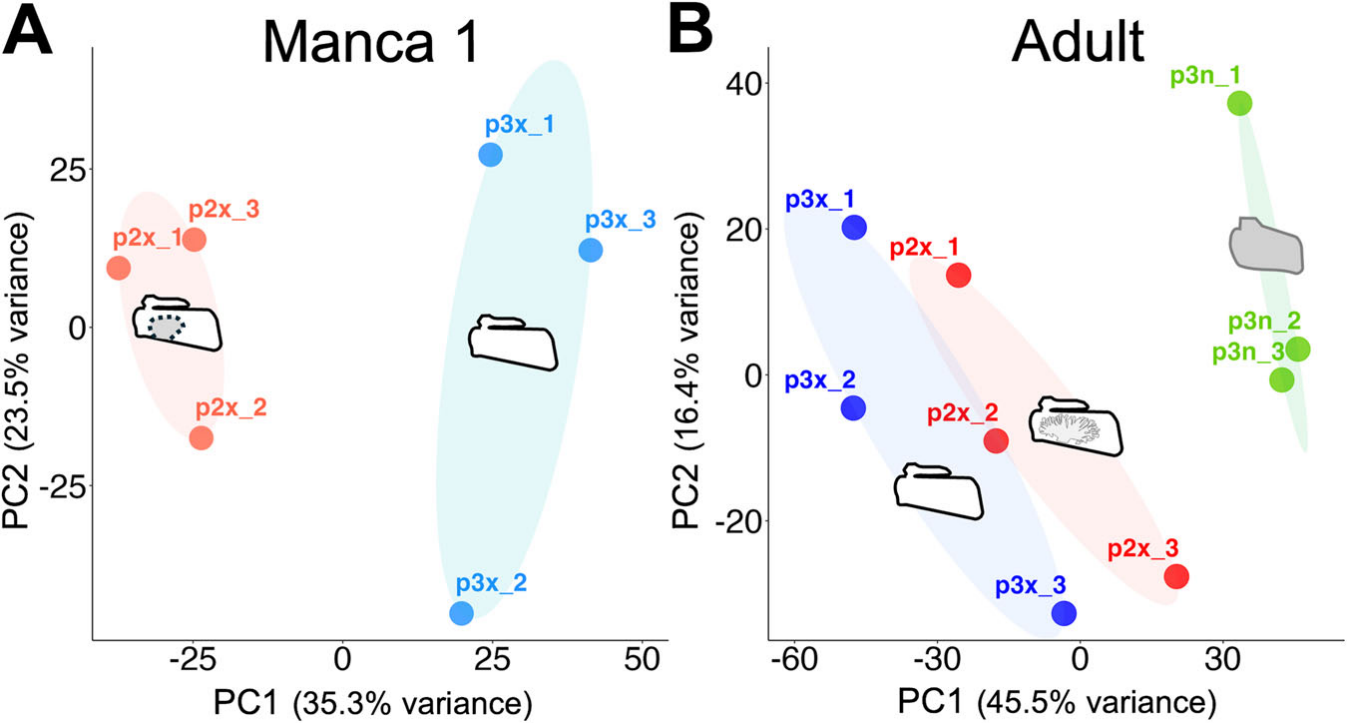
Principal component analysis. (A) Clustering of manca samples. Light red color indicates the exopods of the second pleopods (lungs). Light blue indicates the exopods of the third pleopods (normal exopods). Each dot represents the noted sample. (B) Clustering of adult samples. Red color indicates the exopods of the second pleopods (lungs). Blue indicates the exopods of the third pleopods (normal exopods). Green indicates the endopods of the third pleopods (endopodal gills). PC1, first principal component; PC2, second principal component; p2x, the exopods of the second pleopods; p3n, the endopods of the third pleopods; p3x, the exopods of the third pleopods. [Color figure can be viewed at wileyonlinelibrary.com]

PCA showed that the samples had different expression patterns depending on their biological background (Figure 2). For manca samples, samples derived from the second and third pleopods formed different clusters (Figure 2A). For adult samples, samples from exopods showed different expression patterns from samples from endopods (Figure 2B). However, clusters of exopod samples derived from the second and third pleopods could not be clearly distinguished (Figure 2B).

### 3.3 Differentially Expressed Genes

**Figure 3:**
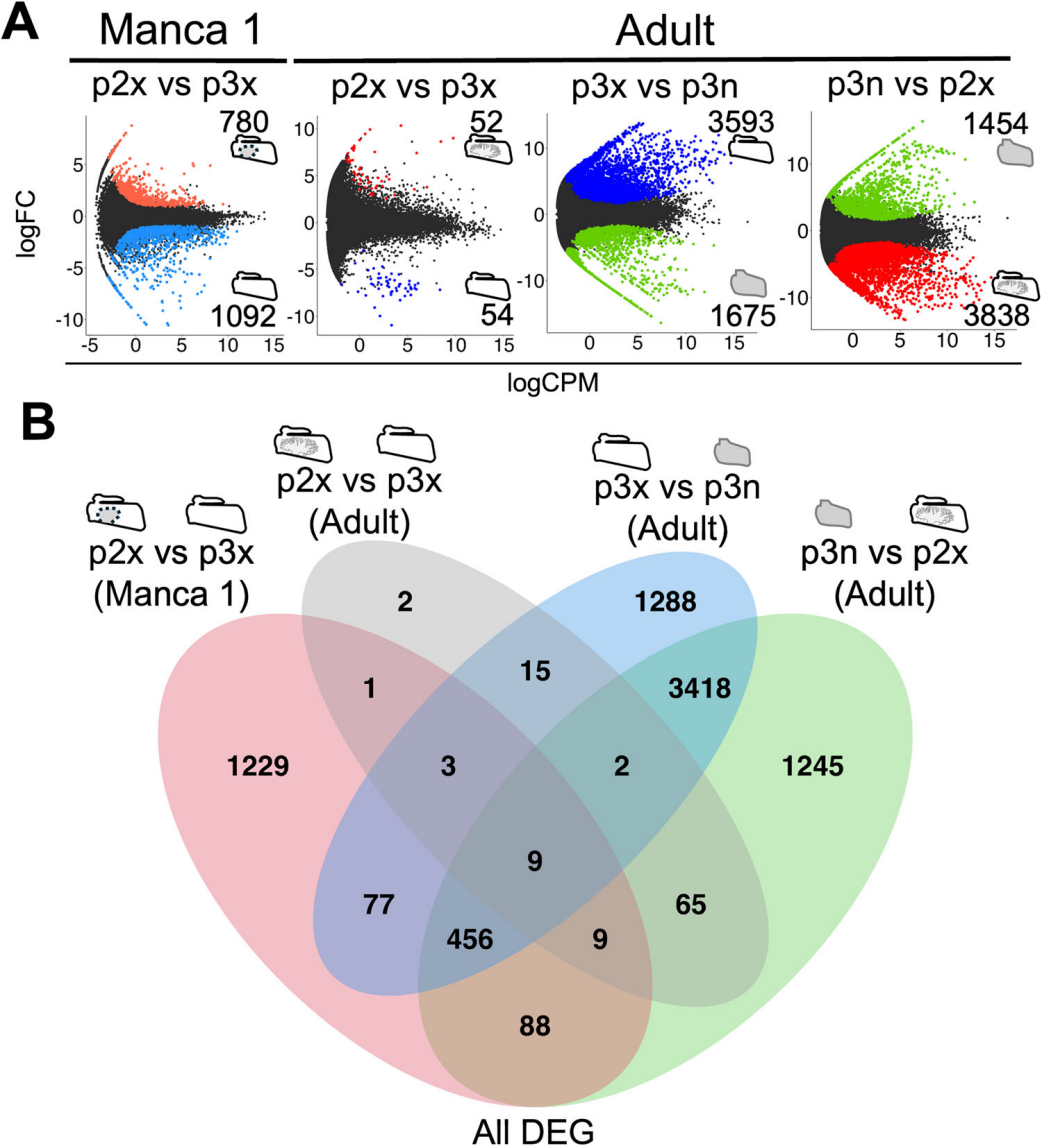
Differential expression analysis. (A) MA plots for all four comparisons. Each dot indicates one contig of superTranscripts and colored dots indicate DEGs (FDR < 0.1). The number of upregulated DEGs in each sample are shown in MA plots. Red color indicates the exopods of the second pleopods (lungs). Blue indicates the exopods of the third pleopods (normal exopods). Green indicates the endopods of the third pleopods (endopodal gills). (B) Venn diagram of all comparisons. Numbers of shared DEGs are shown. DEGs, differentially expressed genes; logCPM, the binary logarithm of counts per million; logFC, the binary logarithm of fold change; p2x, the exopods of the second pleopods; p3n, the endopods of the third pleopods; p3x, the exopods of the third pleopods. [Color figure can be viewed at wileyonlinelibrary.com]

Differentially expressed genes (DEGs) were identified for four pairs of samples: the exopods of second and third pleopods during the manca stage, the exopods of second and third pleopods during the adult stage, the exopods of second pleopods and the endopods of the third pleopods during the adult stage, and the exopods and the endopods of the third pleopods during the adult stage (Figure 3A). The number of DEGs was 1792, 106, 5286, and 5292, respectively (Figure 3: Supporting Information [Link], [Link], [Link], [Link]). The numbers of DEGs shared across different comparisons are shown in Figure 3B.

### 3.4 Comparison Between Pleopodal Exopods and Endopods

**Figure 4:**
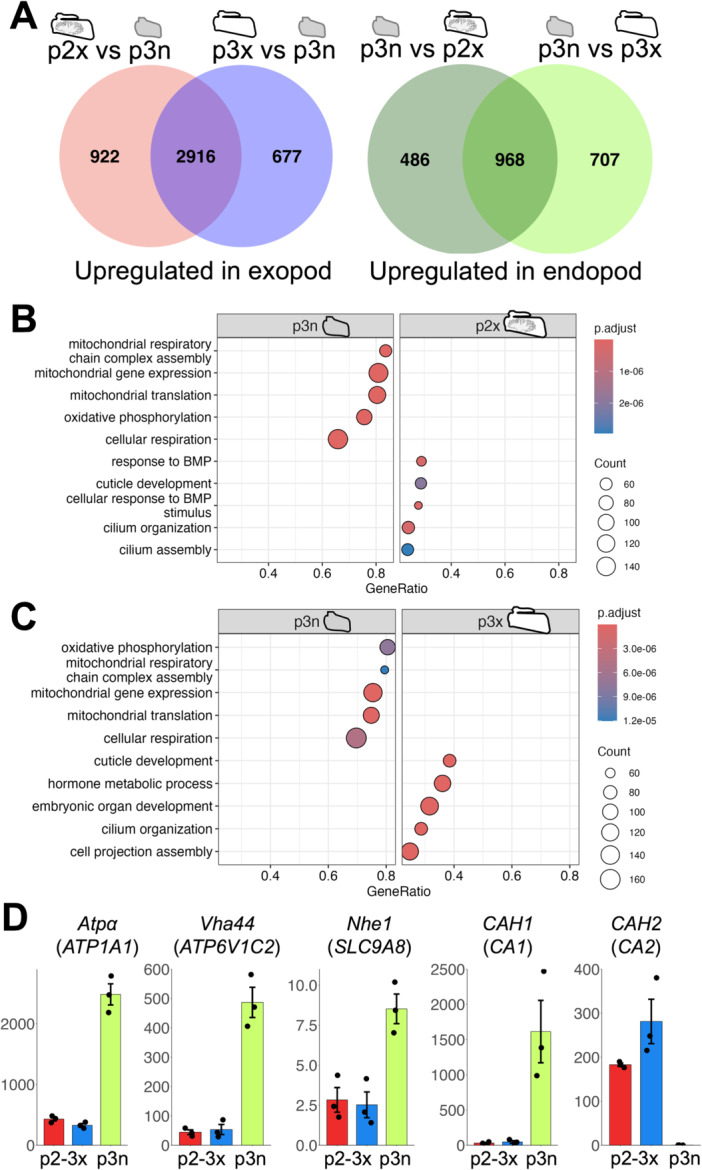
Comparison between pleopodal exopods and endopods. (A) Venn diagrams of DEGs upregulated in exopods and endopods. (B, C) Enriched gene ontology terms of biological function in two comparisons between exopods and endopods. P values and number of genes are shown in tables. The five terms with the highest NES values are shown as representative examples. Whole results are shown in supporting Information [Link], [Link]. (D) TPM values of five genes (*Na pump alpha subunit*, *Vacuolar H[+] ATPase 44kD subunit*, *Na[+]/H[+] hydrogen exchanger 1, Carbonic anhydrase 1*, and *Carbonic anhydrase 2*) related to ion regulation. Dots indicate the TPM value of three biological replicates. Error bars indicate standard error. Gene names based on different annotation methods are listed together. *CAH1*, *Carbonic anhydrase 1*; *CAH2*, *Carbonic anhydrase 2*; *Nhe1*, *Na[+]/H[+] hydrogen exchanger 1*; *Atpα*, *Na pump alpha subunit*; p2x, the exopods of the second pleopods; p3n, the endopods of the third pleopods; p3x, the exopods of the third pleopods; *Vha44*, *Vacuolar H[+] ATPase 44kD subunit*. [Color figure can be viewed at wileyonlinelibrary.com]

To detect genes related to functional differentiation and maintenance of bifurcated pleopods, the differences in gene expression patterns between the exopods and endopods were examined in adult samples (Figure 4).

Among the transcripts that were highly expressed in the exopods compared to the endopods, 2916 transcripts were common between the pairs (Figure 4A: Supporting Information [Link], [Link]). This was 76.0% of the transcripts highly expressed in the exopods of the second pleopods and 81.2% of the transcripts highly expressed in the exopods of the third pleopods. On the other hand, 968 transcripts were shared between the highly expressed transcripts in the endopods compared to the exopods of the second and third pleopods (Figure 4A). This was 66.6% of the transcripts highly expressed in the endopods compared to the exopods of the second pleopods and 57.8% of the transcripts highly expressed in the endopods compared to the exopods of the third pleopods.

Next, to understand the character of genes that were expressed differently between the exopods and endopods, gene set enrichment analyzes were performed (Figure 4B–C: Supporting Information S8). The enriched gene ontology terms were shared between the two pairs. Terms related to BMP signaling, cuticle, and cilia were highly enriched in the exopods of both the second and third pleopods (Figure 4B–C: Supporting Information S8). Terms related to mitochondrial translation, ATP synthesis and respiration were highly enriched in the endopods compared to the exopods (Figure 4B–C: Supporting Information S8).

Finally, based on previous studies focusing on crustacean gill functions (Ali et al. 2015; Tsai and Lin 2007), five representative genes related to ion regulation were explored and their expression patterns were examined (Figure 4D). Four of them (*Na pump alpha subunit*, *Vacuolar H[+] ATPase 44kD subunit*, *Na[+]/H[+] hydrogen exchanger 1, Carbonic anhydrase 1*) tended to be highly expressed in the endopod of the third pleopod (Figure 4D). Among the DEGs, other ion exchangers were also highly expressed in the endopods (Supporting Information [Link], [Link]).

On the other hand, *Carbonic anhydrase 2* was highly expressed in the exopods (Figure 4D: Supporting Information [Link], [Link]).

### 3.5 Candidate Genes for Lung‐Specific Expression

**Figure 5:**
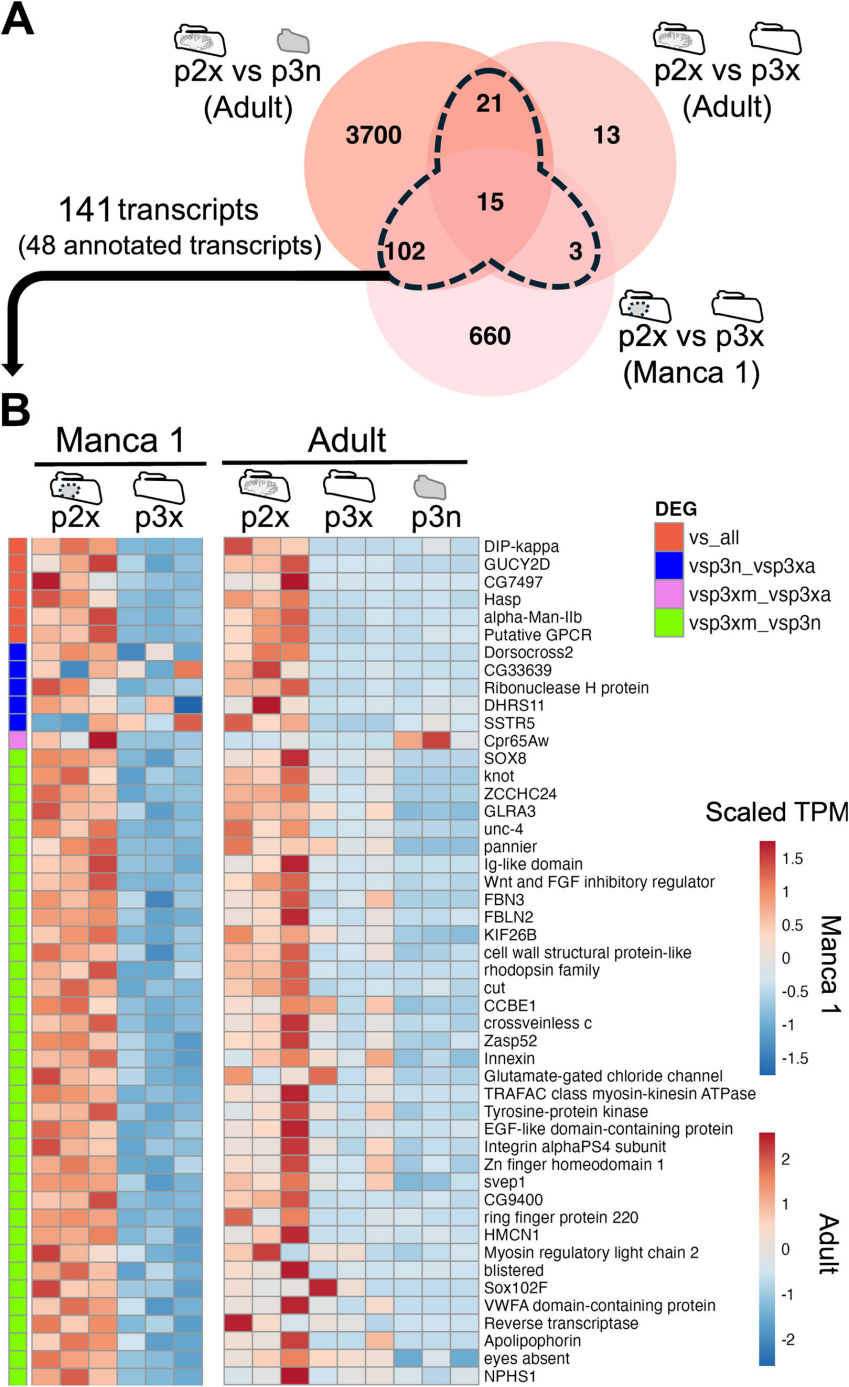
Candidate lung‐specific genes. (A) Venn diagram of DEGs upregulated in the exopods of the second pleopods among three comparisons. An area surrounded by dotted line indicate shared DEGs (candidate lung‐specific genes). (B) Heatmap of candidate lung‐specific genes shows scaled expression patterns. The leftmost colored columns show the position in which each transcript is included in the Venn diagram (A). The gene names are one of the names given by different annotation methods. TPM, transcripts per million; p2x, the exopods of the second pleopods; p3n, the endopods of the third pleopods; p3x, the exopods of the third pleopods. [Color figure can be viewed at wileyonlinelibrary.com]

To investigate the molecular background of lung structure and function in *P. scaber*, transcripts that are specifically expressed in the lung were explored (Figure 5).

Among the comparisons including the exopods of the second pleopods, 141 DEGs were partly or fully common with other pairs (Figure 5A, area surrounded by dotted line in the Venn diagram: Supporting Information S4, S5, and S7). Among these 141 shared DEGs, 48 transcripts were annotated (Figure 5B). These were defined as candidate lung‐specific genes. Six transcripts were upregulated in the endopod in all 3 comparisons: *Dpr‐interacting protein kappa* (*Lachesin*) (Supporting Information S3), *guanylate cyclase 2D*, *Drosophila* gene *CG7497*, *Hig‐anchoring scaffold protein*, *alpha‐Mannosidase class II b*, and putative *G‐protein coupled receptor*. Five transcripts were expressed in both the adult exopods and endopods of the third pleopods: *Dorsocross2, Drosophila* gene *CG33639, Ribonuclease H protein, Dehydrogenase/Reductase 11, Somatostatin receptor type 5*. One transcript (*Cuticular protein 65Aw*) (Supporting Information S3) was highly expressed compared to its expression level in the exopods of the third pleopods in adult and manca samples. The other 36 transcripts were comparably expressed relative to their expression levels in the exopods of the third pleopods in manca and the adult endopods (Figure 5B).

**Figure 6:**
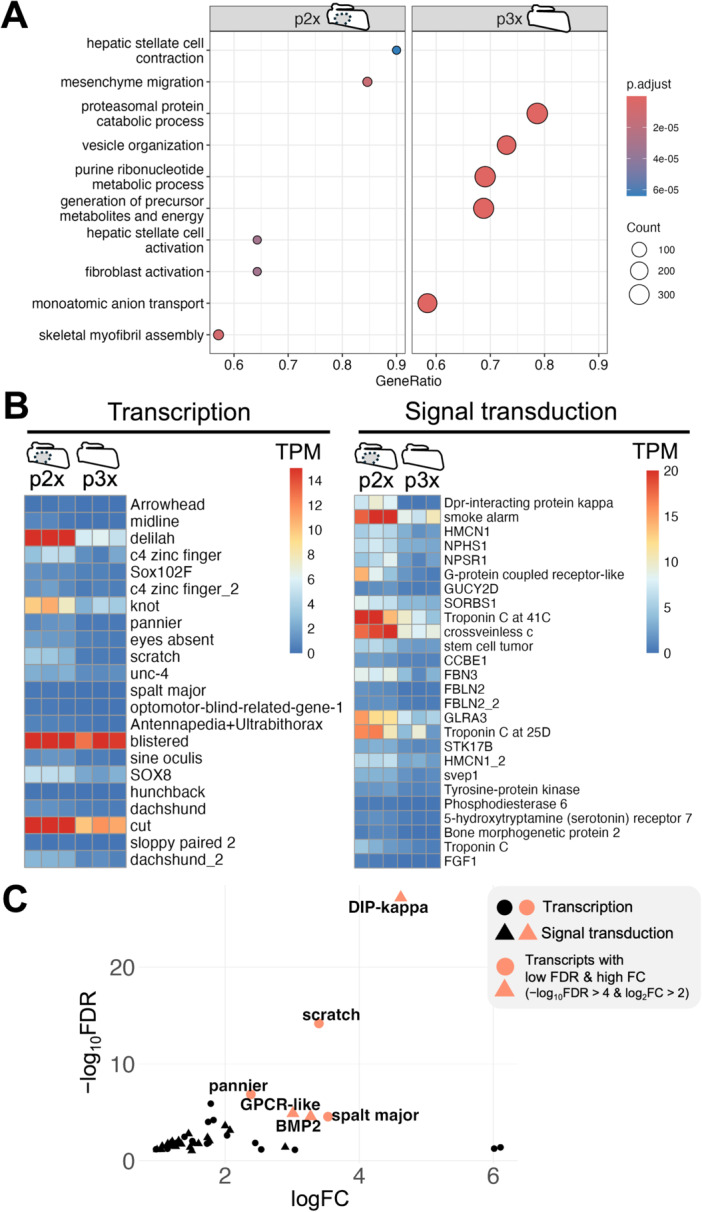
Genes related to early lung development. (A) Enriched gene ontology terms of biological function in a comparison between the exopods of the second and third pleopods. *p* values and number of genes are shown in tables. The five terms with the highest NES values are shown as representative examples. Whole results are shown in supporting information S8. (B) Heatmaps of DEGs with transcription or signal transduction role show the TPM value of each DEG. All TPM values over 15 (transcription) and 20 (signal transduction) are shown as the same red color. Gene names are one of the names given by different annotation methods. (C) One‐sided volcano plot of upregulated DEGs with transcription (circle) or signal transduction (triangle) role. Genes with a tendency to exhibit high differential expression are shown in orange. log_10_FDR, the common logarithm of false discovery rates; logFC, the binary logarithm of fold change; TPM, transcripts per million; p2x, the exopods of the second pleopods; p3x, the exopods of the third pleopods. [Color figure can be viewed at wileyonlinelibrary.com]

In addition, to gain insights into the mechanisms of early lung morphogenesis, DEGs for manca pleopods were examined in detail (Figure 6: Supporting Information S4). Gene set enrichment analysis was conducted for DEGs of manca samples (Figure 6A: Supporting Information S8). Gene ontology terms related to hepatic cells, cytoskeleton, and cell migration were enriched for DEGs that were upregulated in the exopods of the second pleopods (Figure 6A). On the other hand, terms related to metabolic processes and ion transport were enriched for DEGs that were upregulated in the exopods of the third pleopods (Figure 6A).

Moreover, to search for genes involved in the regulation of early lung development, transcripts with the function of transcription or signal transduction were listed among the upregulated DEGs in the exopods of the second pleopods using Clusters of Orthologous Genes (COG) category codes (Figure 6B–C) (Tatusov et al. 2003). Focusing on transcripts per million (TPM) values, transcription factors (COG category: K) such as *delilah, knot, blistered* and *cut* showed relatively high expression levels in the manca stage (Figure 6B). Regarding signaling molecules (COG category: T), genes such as *smoke alarm, Troponin C at 41 C, crossveinless C, Glycine receptor subunit alpha‐3* and *Troponin C at 25D* showed relatively high expression levels in the manca stage (Figure 6B). Conversely, focusing on FDR and fold change values revealed relatively strong differential expression of transcription factors *pannier*, *scratch*, and *spalt major*, as well as signaling molecules *Dpr‐interacting protein kappa* (*Lachesin*), putative *G‐protein coupled receptor*, and *bone morphogenetic protein 2* showed relatively strong differential expression (Figure 6C).

### 3.6 Hox Gene Expression in Abdomen

**Figure 7:**
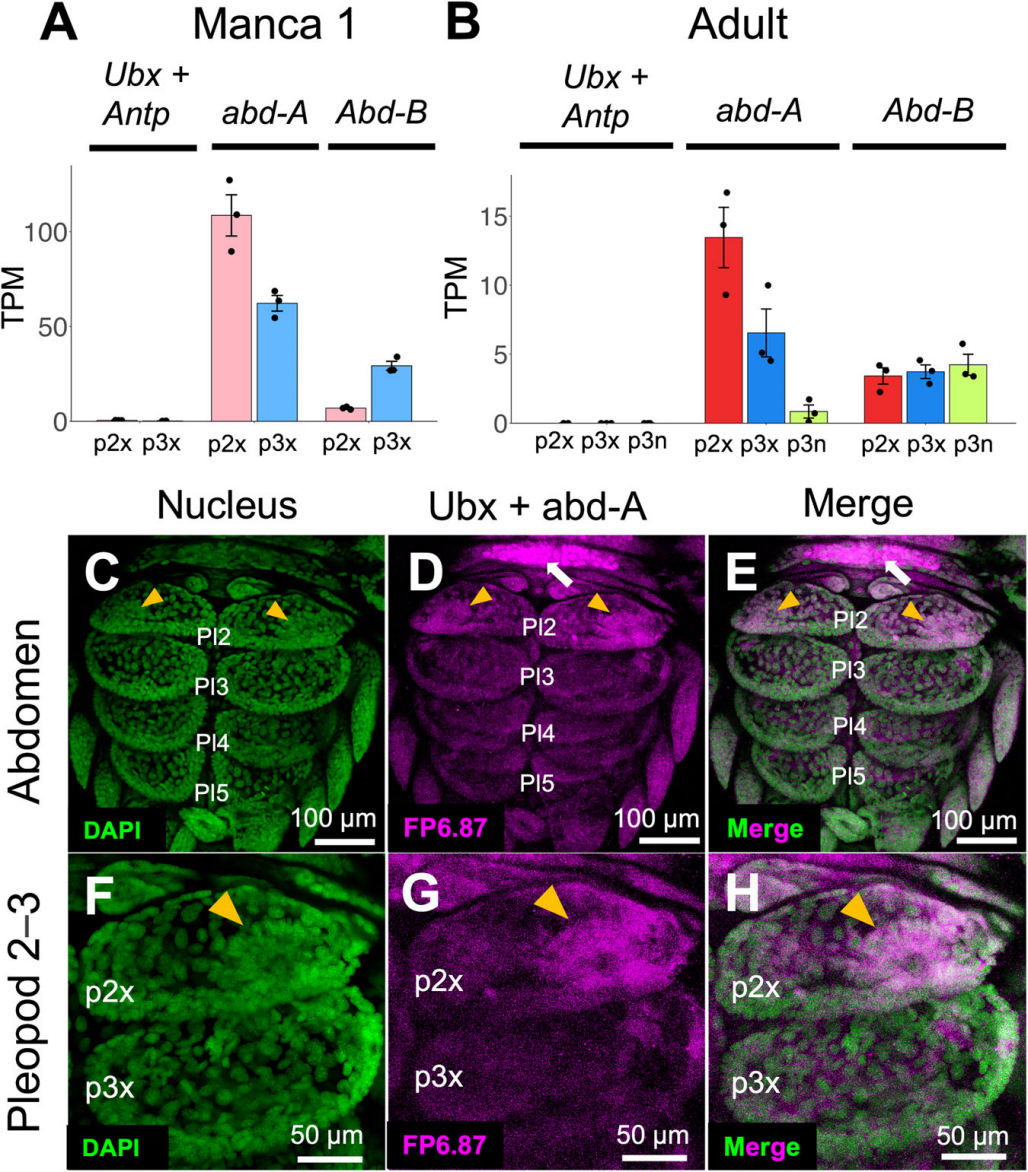
Hox gene expression in the abdomen. (A, B) TPM values of *Ubx*+*Antp*, *abd‐A* and *Abd‐B* in manca (A) and adult (B) samples. Dots indicate the TPM value of three biological replicates. Error bars indicate standard error. (C–H) Immunofluorescence of Ubx and/or Abd‐A in an abdomen of *P. scaber* at manca 1 stage. DAPI staining of nucleus (C, F), Ubx and Abd‐A expression detected by FP 6.87 (D, G), and merge of these (E, H) are shown. Orange arrowheads indicate cell aggregation and strong expression in the exopods of the second pleopods. White arrows indicate strong expression in the ventral side of the thoracic segment. Gene names for each contig are represented by the names of the homologous genes in *D. melanogaster*. Pl, pleopod; p2x, the exopods of the second pleopods; p3x, the exopods of the third pleopods. [Color figure can be viewed at wileyonlinelibrary.com]

To gain insight into the mechanism of patterning of the different kinds of pleopods, the expression patterns of central or posterior Hox genes (*antennapedia* (*Antp*), *Ultrabithorax (Ubx)*, *abdominal‐A*, and *Abdominal‐B* (*Abd‐B*)) were explored and examined based on previous research (Figure 7) (Abzhanov and Kaufman 2000a, 2000b; Brena et al. 2005; Tan and Monteiro 2025). In the superTranscripts of *P. scaber* created in this study, *Antp* and *Ubx* are treated as a single fusion gene due to their bicistronic transcription (Shiga et al. 2006).

Although *Antp* + *Ubx* was detected as a DEG in manca samples (Figure 6B: Supporting information S4), the TPM values of the sum of *Antp* and *Ubx* were less than 1 (Figure 7A–B). *Abd‐A* was expressed more highly in the exopods than in the endopods for the adult sample (Figure 7A). Although it was not detected as a DEG (FDR < 0.1) for the manca sample, it tended to be highly expressed in the exopods of the third pleopods compared to those of the second exopods (Figure 7B). *Abd‐B* did not show differential expression among pleopods of the adult samples, while it was expressed more highly in the exopods of the second pleopods than those of the third pleopods for the manca sample (Figure 7A–B: Supporting Information S4).

The expression of either Ubx or Abd‐A, or both in the second pleopods was detected using immunofluorescence with FP6.87, which recognizes both Abd‐A and Ubx. (Figure 7C–H). There were differences in the cell arrangements between the exopods of the second and third pleopods. Cells were aggregated at the edge of exopods of the second pleopods (Figure 7C–H; arrowheads). Although Ubx or/and Abd‐A were weakly expressed in the whole abdomen, these proteins were strongly expressed at the aggregated cells in the edge of the exopods of the second pleopod (Figure 7G, H; arrowheads). In addition, cells with strong expression were observed in the ventral side of the thoracic segment (Figure 7D, E; arrows).

### 3.7 Expression Pattern of Appendage Patterning Genes

**Figure 8:**
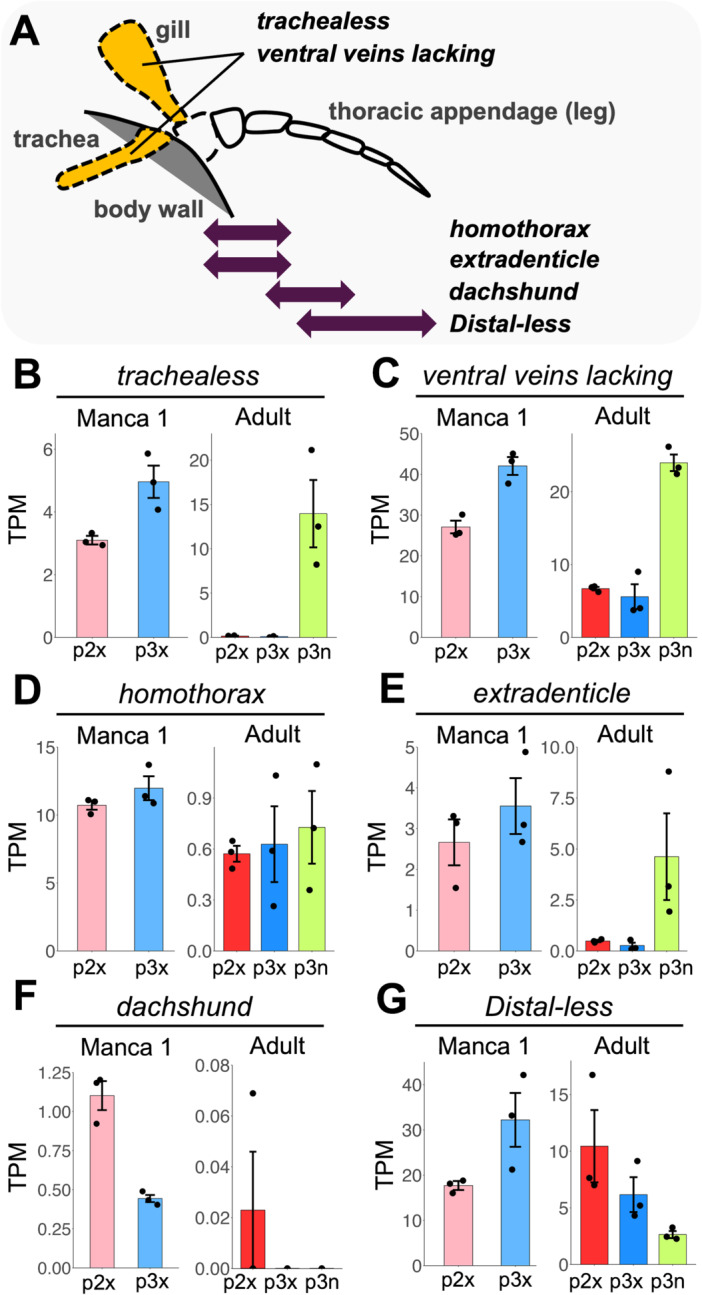
Expression pattern of appendage patterning genes. (A) Appendage patterning genes focused on in this study. The scheme of a model of a pancrustacean thoracic appendage. In existent animals, the tracheal system and epipodal gills do not exist in the same appendage. Gene expression patterns are based on previous studies (Abzhanov and Kaufman 2000b; Bruce 2021; Franch‐Marro et al. 2006) and shown in orange color (*trachealess, ventral veins lacking*) and purple two‐way arrows (*homothorax, extradenticle, dachshund, Distal‐less*). (B–G) TPM values of appendage patterning genes. Dots indicate the TPM value of three biological replicates. Error bars indicate standard error. Gene names for each contig are represented by the names of the homologous genes in *D. melanogaster*. TPM, transcripts per million; p2x, the exopods of the second pleopods; p3x, the exopods of the third pleopods. [Color figure can be viewed at wileyonlinelibrary.com]

The expression patterns of transcription factors involved in the formation of respiratory organs or appendages in arthropods were then investigated (Figure 8). Two transcription factors related with respiratory organs (*trh* and *vvl*) and four transcription factors involved in appendage patterning (*homothorax* (*htx*), *extradenticle* (*exd*), *dachshund* (*dac*), *Distal‐less* (*dll*)) were identified in the superTranscripts of *P. scaber* (Abzhanov and Kaufman 2000b; Bruce 2021; Hanna and Popadić 2020; Tan and Monteiro 2025) (Figure 8A).

The former two genes, *trh* and *vvl*, showed similar expression patterns (Figure 8B,C): they were more highly expressed in the endopods than in the exopods in adult samples (Supporting Information [Link], [Link]), while there were no significant differences between the exopods of the second and third pleopods. Although they were not detected as DEGs for the manca sample (Supporting information S4), *trh* and *vvl* tended to be highly expressed in the exopods of the third pleopods compared to those of the second pleopods.

*Hth* and *exd* were not detected as DEGs (Supporting Information [Link], [Link], [Link], [Link]). However, in adults, *exd* tended to be highly expressed in endopods compared to exopods.

*dac* was detected as a DEG in mancas (Supporting Information S4) and highly expressed in the exopods of the second pleopods (Figure 8F). However, its TPM value was relatively lower than those of other transcription factors. In adults, *dac* expression was not detected in any samples except one (Figure 8F).

*Dll* was detected as a DEG in adults and highly expressed in the exopods of the second pleopods compared to endopods (Figure 8G).

## 4 Discussion

### 4.1 Reference Gene Catalogs of *P. scaber*

*Porcellio scaber* is a common oniscid species and one of the organisms which are suitable for comparative studies in evolutionary and developmental biology (Wolff 2009). Although the partial genome or transcriptome of this species has already been obtained in previous studies (Becking et al. 2017; Collins et al. 2023), the whole transcriptome including various developmental stages has not been investigated. In this study, the developmental transcriptome was successfully obtained with 97.9% complete BUSCOs, and the comprehensive gene sequences of this species were predicted (Supporting Information S3: Table S2).

Although the rate of complete BUSCOs decreased from 97.9% to 90.9% due to the creation of superTranscripts, the rate of duplicated BUSCOs also decreased significantly from 61.9% to 1.9% (Supporting Information Table S2). This suggested that redundant sequences have been appropriately reduced for comparative RNAseq analysis. In addition to the partial sequences of some genes related to development that were sequenced in previous studies (Abzhanov and Kaufman 2000b; Brena et al. 2005), more than 10,000 gene sequences for this species were obtained in this study (Supporting Information S3).

### 4.2 Differential Gene Expression Patterns Between the Pleopodal Exopods and Endopods

It has been suggested that the exopods of isopod pleopods generally function in gas exchange, while the endopods are primarily involved in osmoregulation (Wägele 1982). The exopods and endopods of isopod pleopods also have different tissue structures (Wägele 1982). A similar functional difference of pleopods is thought to apply to terrestrial isopods (Surbida and Wright 2001). There were thousands of differentially expressed genes between the exopods and endopods in adults, emphasizing the differences in their functions and tissue structures in adults (Figures 2, 3, 4).

It is known that the epithelial cells that are responsible for ion regulation in the gills of crustaceans contain a large number of mitochondria (Freire et al. 2008). Transcripts that are highly expressed in the endopodal gills are mainly related to mitochondrial metabolism and intracellular respiration (Figure 4B–C), suggesting that these parts could be related to the ion regulation in the pleopodal gill chamber (Pequeux 1995; Surbida and Wright 2001). Some ion‐exchangeable genes were highly expressed in endopods (Figure 4D).

In contrast, the transcripts that are highly expressed in the exopods include genes related to cuticle structure formation and morphogenesis (Figure 4B–C). Although it is difficult to directly infer gas exchange capacity in the exopods from gene expression patterns, these patterns may account for the differences in exoskeleton structure. In addition, bone morphogenetic protein signaling is known to be involved in the formation of the dorsal/ventral axis of the whole embryo and the anterior/posterior axis of the appendages during embryonic development (Akiyama et al. 2023). This signaling pathway may be involved in determining or maintaining the identity of the exopods and endopods.

### 4.3 Developmental Mechanisms of Pleopodal Lungs

*Porcellio scaber* developed pleopodal lungs in the exopods of the first two pleopods for air‐breathing (Inui et al. 2022) (Figure 1). In adults, the difference between the gene expression patterns of the lung‐bearing exopods and lungless exopods was smaller than the difference between the expression patterns of the exopods and endopods (Figures 2, 3). Although the DEGs upregulated in the adult lung‐bearing exopods included some genes probably related to the lung structure (*Dpr‐interacting protein kappa* (*Lachesin*) and *Cuticular protein 65Aw*) (Figure 5: Supporting Information S3), there were few upregulated DEGs that are involved in development (Figure 6).

Although it is difficult to make a statistical comparison with adults due to differences in the sequencing methods, more genes showed differential expression in the manca 1 stage (Figure 5). The upregulated DEGs in lung‐bearing exopods include genes involved in morphogenesis, such as the formation of the cytoskeleton, and may be involved in the initial development of the lung (Figures 5, 6; Supporting Information S5). These expression patterns suggest that the gas exchange ability in the pleopodal lungs could be mainly established by exoskeletal and epithelial structures developed during the postembryonic stages.

Among the upregulated DEGs in lung‐bearing exopods of the manca sample (Figure 6: Supporting Information S4), candidate transcription factors and signaling molecules that were possibly involved in lung development were identified (Figure 6B–C). Moreover, the expression patterns of Hox genes and several transcription factors that have been implicated in lung development (Bruce 2021; Tan and Monteiro 2025) were also examined (Figures 7, 8).

*Ubx+Antp* and *Abd‐B* was detected as DEGs at manca 1 stage and the expression patterns of Hox genes differed between the pleopods. The expression patterns of Hox genes were generally consistent with those during embryonic development (Abzhanov and Kaufman 2000a; Brena et al. 2005). However, for *Abd‐B*, there was almost no difference in the expression pattern between the pleopods in the adult (Figure 7B). Although it was not detected as a DEG, *abd‐A* is highly expressed in the second pleopods throughout the developmental stage, and this gene may contribute to lung formation. In addition, *Abd‐B* is highly expressed in the third pleopods at the manca stage (Figure 7A), and it is possible that this gene contributes to the suppression of lung formation. The high expression of *abd‐A* in the exopods of the second pleopods during the manca stage was also confirmed by immunofluorescence (Figure 7C–H). The detected expression reflects the total amount of Ubx and Abd‐A, but considering that Ubx is hardly expressed in the pleopods (Figure 7A), it is suggested that the fluorescence in the abdominal leg was due to the expression of Abd‐A. The location of the aggregated cells coincides with the developing spiracle of the lung (Inui et al. 2022), suggesting the presence of specific epithelial cells that form the lung and the initial formation of the lung through cell migration and exoskeleton formation. The formation of differences in the structure of adjacent abdominal segments is likely due to these Hox genes.

On the other hand, *trh* and *vvl*, which are thought to be involved in respiratory organ formation in pancrustaceans (Tan and Monteiro 2025), were not included in the DEGs at manca 1 stage (Supporting Information S4), and did not show specific expression in the exopods of the second pleopods (Figure 8B–C). Based on the previous study (Abzhanov and Kaufman 2000b) and the adult expression pattern (Figure 8B–C: Supporting Information S3–S4), it could be assumed that these genes regulate endopodal gill development rather than exopodal lungs.

Regarding other appendage patterning genes, *dac* and *dachshund_2* showed lung‐related expression patterns (Figure 6B: Figure 8F). DEGs in the manca comparison also included *eyes absent* and *sine oculis* genes and these imply the involvement of a Pax‐Six‐Eya‐Dach network (Donner and Maas 2004). However, the expression levels of these genes were relatively low compared to those of other DEGs (Figure 6B).

**Figure 9:**
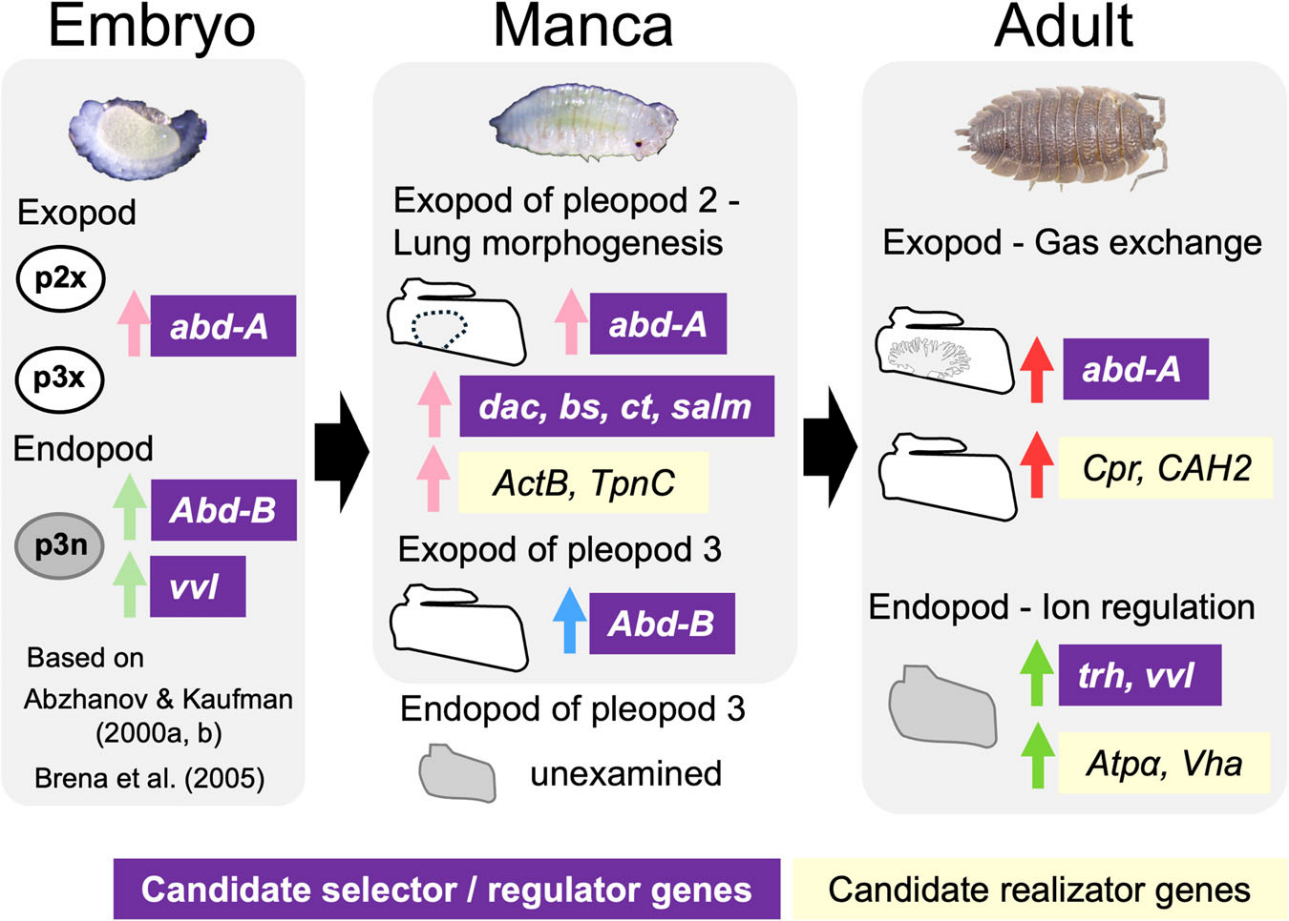
Hypothetical molecular landscape of pleopodal lung development in *P. scaber*. Gene names and arrowheads indicate upregulation of candidate genes in specific pleopods. Gene expression patterns during embryonic development were according to the previous studies (Abzhanov and Kaufman 2000a; Abzhanov and Kaufman 2000b; Brena et al. 2005). p2x, the exopods of the second pleopods; p3x, the exopods of the third pleopods. [Color figure can be viewed at wileyonlinelibrary.com]

Finally, the possible regulators of lung development are discussed (Figure 6B–C: Figure 9). Given the low likelihood of involvement of *trh* and *vvl*, it is presumed that there are other upstream genes that regulate lung development. The candidate DEGs include genes related to appendage morphogenesis or epithelial tissue formation (Figure 6B–C). For example, transcription factors *blistered*, *knot* or *cut* showed high TPM values and *spalt major* showed strong differential expression. They are involved in the formation of insect wings (Fristrom et al. 1994; Macdonald et al. 2010; de Miguel et al. 2016), which were suggested to be homologous to crustacean epipodal gills (Bruce and Patel 2020; Clark‐Hachtel and Tomoyasu 2020). Especially, the transcription factor *cut* is also known to be involved in the formation of the insect spiracle (Hanna and Popadić 2020) and the formation of gills in amphipods (Liu et al. 2023). However, *blistered* or *cut* also showed a certain level of expression in the exopods of the third pleopods (Figure 6B; Supporting information S4), it will be necessary to investigate their detailed localization in the exopods of the second pleopods in a future study.

Based on the results of the DEGs and gene set enrichment analyzes, it is possible to hypothesize a model in which genes that contribute to cytoskeleton formation are expressed at higher levels downstream of these candidate regulatory factors, and that those downstream genes cause the migration and adhesion of epithelial cells. However, as these gene sets also contribute to the morphogenesis of other organs (Jinushi‐Nakao et al. 2007), to investigate the contribution of these genes to lung development, it will be necessary to perform functional analysis of candidate genes in the future.

## 5 Conclusion and Future Perspectives

In this study, comprehensive gene expression patterns of various kinds of pleopods, including lung‐bearing exopods, normal exopods and endopods, in a terrestrial isopod were obtained. Based on these results, possible candidate genes involved in pleopodal lung development were identified (e.g., *dac*, *blistered*, *knot*, *cut* or *spalt major*). To clarify the developmental mechanism of the isopod pleopodal lung, it will be necessary to track cells expressing candidate genes using fluorescent labels and to perform functional analysis of the candidate genes. Furthermore, it will be possible to infer the evolutionary processes of the pleopodal lung by examining the expression dynamics of candidate genes in basal terrestrial isopods that lack pleopodal lungs.

## Author Contributions

Naoto Inui and Toru Miura designed the study. Naoto Inui collected and kept material animals. Naoto Inui and Sumio Udagawa performed the dissection and RNA extraction. Naoto Inui, Akifumi Yao, Kohei Oguchi, Sumio Udagawa, Yoshinobu Hayashi, and Toru Miura analyzed the RNA‐seq results. Naoto Inui performed immunofluorescence analysis. All authors wrote the article and approved the final version of the article.

## Conflicts of Interest

The authors declare no conflict of interest.

## Supporting information

Supporting_information_Table S1. Supplemental information for each read.

Supporting information_Table S2. BUSCO results.

Supporing information_S3. Annotated contigs in the superTranscripts of *P. scaber*. (A) Number of contigs in analysis. (B) Venn diagram of annotated contigs. Numbers of shared annotated contigs are shown. Figure S2 Examples of lung‐specific DEGs involved in downstream biological processes. (A) *DIP‐kappa* (*Lachesin*). (B) C*uticle protein 65Aw*.

Supporting_information_S4.

Supporting_information_S5.

Supporting_information_S6.

Supporting_information_S4.

Supporting_information_S8.

Supporting_information_S9.

Supporting_information_S10.

Supporting_information_S11.

## Data Availability

Datasets used in this study have been deposited to NCBI/DDBJ/EMBL and Zenodo. Raw sequence reads and assembled contigs generated using Trinity have been deposited in NCBI/DDBJ/EMBL database under the BioProject accession number PRJDB20244. Created superTranscripts file, a table of correspondence between Trinity assembly files and superTranscripts, and annotation information of superTranscripts is available on Zenodo database (doi. org/10.5281/zenodo.15011027).
