# Supplementary material for: Gene Expression Analysis Provides Insights Into the Functional and Developmental Differentiations of Pleopodal Lungs in a Terrestrial Isopod Crustacean, Porcellio scaber
Source: Evol Dev. 2025 Nov 29;27(4):e70026. doi: 10.1111/ede.70026 (PMC12664292; doi:10.1111/ede.70026)
Supplement: Supplementary file 3 — Supporing information_S3. Annotated contigs in the superTranscripts of P. scaber. (A) Number of contigs in analysis. (B) Venn diagram of annotated contigs. Numbers of shared annotated contigs are shown. Figure S2 Examples of lung‐specific DEGs involved in downstream biological processes. (A) DIP‐kappa (Lachesin). (B) Cuticle protein 65Aw. [file EDE-27-e70026-s011.docx]

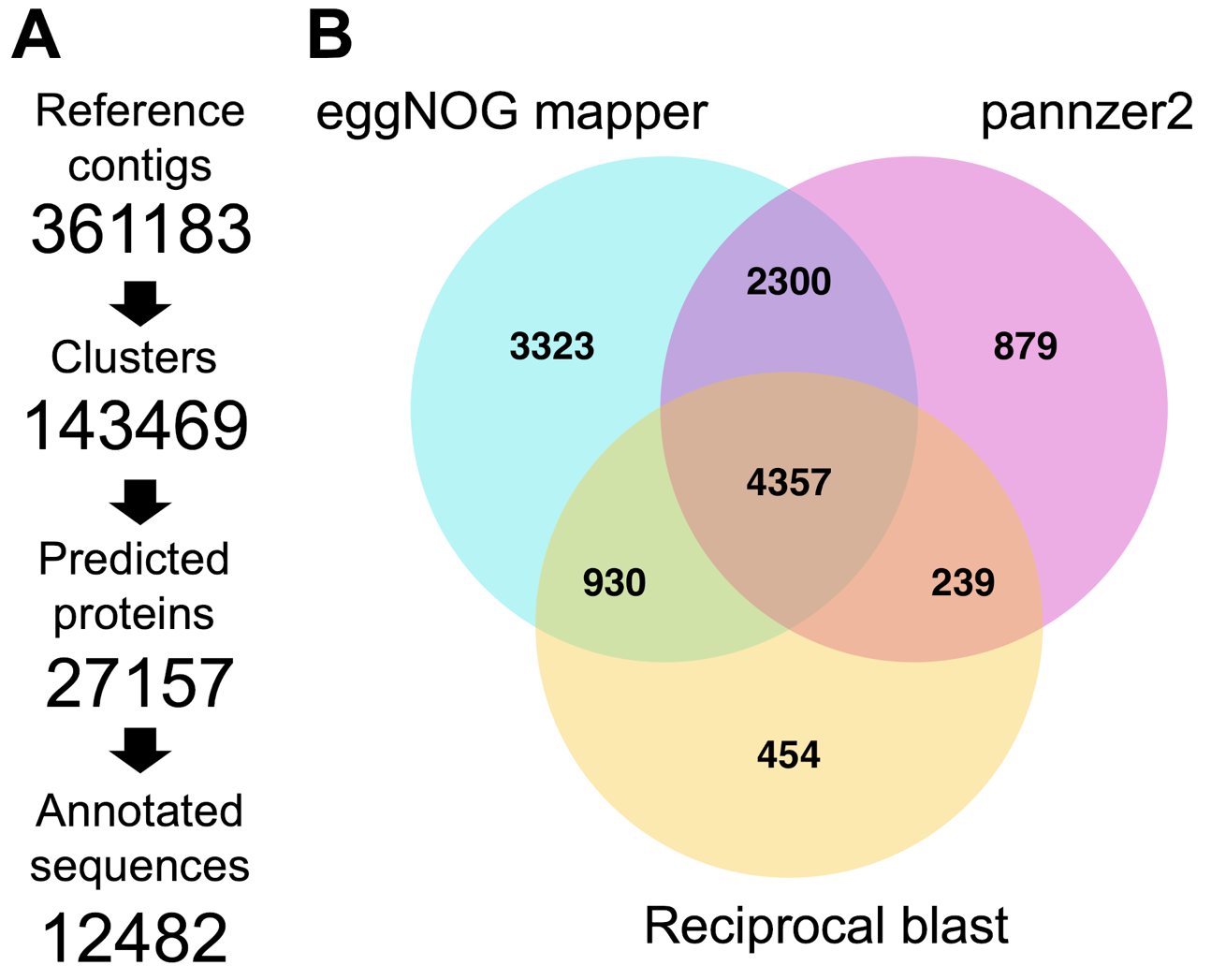


Figure S1

Annotated contigs in the superTranscripts of *P. scaber*. (A) Number of contigs in analysis. (B) Venn diagram of annotated contigs. Numbers of shared annotated contigs are shown.


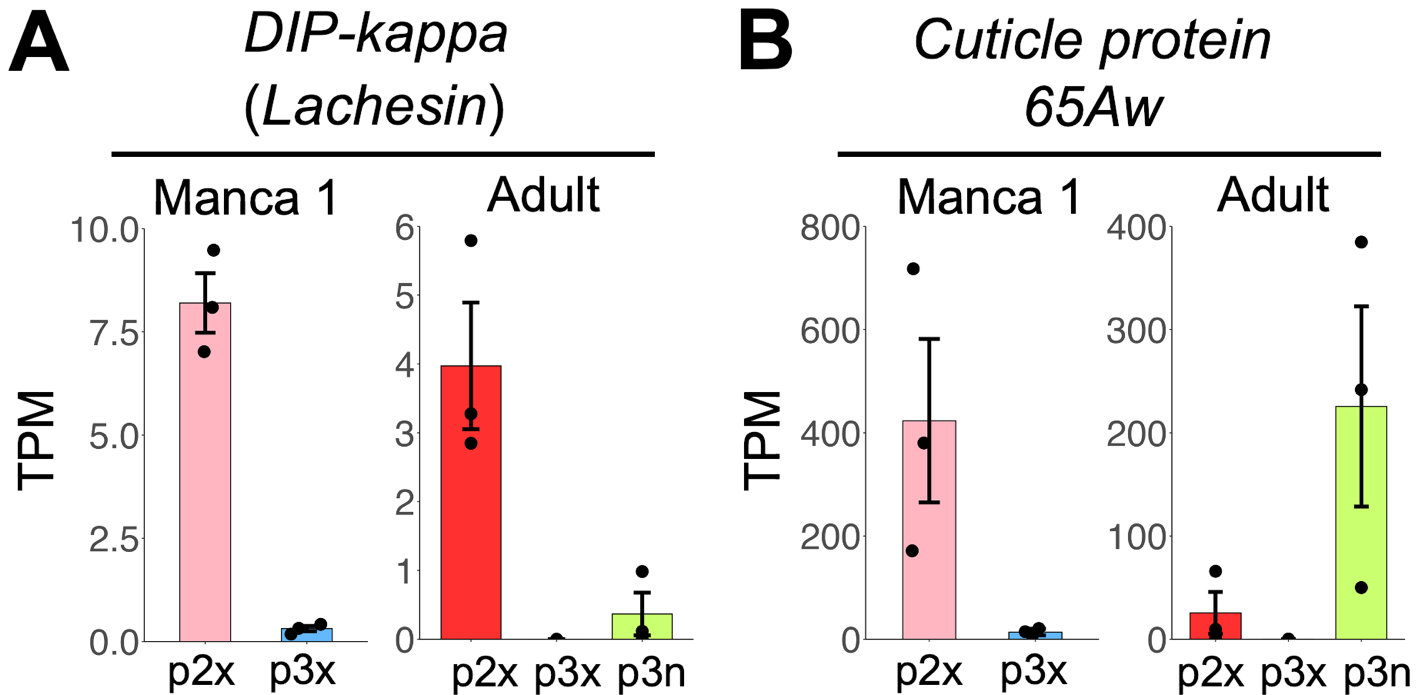


Figure S2

Examples of lung-specific DEGs involved in downstream biological processes. (A) *DIP-kappa* (*Lachesin*). (B) C*uticle protein 65Aw*. Abbreviations: TPM: transcripts per million; p2x: the exopods of the second pleopods; p3n: the endopods of the third pleopods; p3x: the exopods of the third pleopods.
